# Draft genome report of *Vibrio alginolyticus* from marine water of Saint Martin Island, Bangladesh

**DOI:** 10.1128/mra.01290-24

**Published:** 2025-11-10

**Authors:** Fahad Khan, Abdus Sadique, Abdullah All Jaber, Md. Sakib Abrar Hossain, Rayhan Imam, Md. Mashiur Rahman, Jahidul Alam, Raiyan Rafsan, Katha Saha, Md. Azmain Faik, Taposh Kumar Das, Oly Ahmed, Fariza Shams, Ayesha Sharmin, Md. Mainul Hossain, Kamruzzaman Rumman, Munirul Alam, Maqsud Hossain

**Affiliations:** 1 NSU Genome Research Institute (NGRI), North South University 54495, Dhaka, Bangladesh; 2 Department of Biochemistry & Microbiology, School of Health and Life Sciences, North South University 54495, Dhaka, Bangladesh; 3 Department of Electrical and Computer Engineering, School of Engineering and Physical Sciences, North South University 732508 , Dhaka, Bangladesh; 4 Invent Technologies Limited, Dhaka, Bangladesh; 5 Department of Chemistry, Bangladesh University of Engineering and Technology 61750, Dhaka, Bangladesh; 6 National Institute of Cancer Research and Hospital (NICRH) 332897, Dhaka, Bangladesh; 7 International BioScience and Oncology Research Limited (IBSOR), Dhaka, Bangladesh; 8 icddr, b (International Centre for Diarrheal Disease Research, Bangladesh), Dhaka, Bangladesh; California State University San Marcos, San Marcos, California, USA

**Keywords:** *vibrio alginolyticus*, antibiotic resistance, virulence genes, public health risk, environmental monitoring

## Abstract

*Vibrio alginolyticus* strain NGCXV-111A, isolated from Saint Martin Island, Bangladesh displays genomic traits with potential implications for environmental and public health. Genomic analysis identified key virulence genes (*vscF, vscI, vopR, and tlh*), emphasizing the need for ongoing monitoring of Bangladesh’s marine environments to safeguard public health and ecological balance.

## ANNOUNCEMENT

Saint Martin Island is home to diverse marine species, including corals and seaweeds, making it a vital biodiversity hotspot (1). It faces significant challenges from human activities, unregulated tourism, and environmental changes (2). Monitoring microbial species like *Vibrio alginolyticus* is essential, as this gram-negative, halophilic bacterium harbors antimicrobial resistance and virulence genes that may threaten aquaculture, coral health, and human health, though further functional validation is required (3).

On 16 December 2022, both seawater (**~**500 mL) and surface sediment (collected from ~150 mm depth) were sampled from Chhera Dwip, Saint Martin Island, Bangladesh (20°34′53.8″ N, 92°20′16.8″ E). Water samples were stored in sterile bottles (Nalgene, USA), and sediment samples in polyethylene bags, transported at 4–8°C, and processed within 24 h. For enrichment, 1 mL seawater was added to 9 mL Bile Peptone Water (BPW; HiMedia, India; 10 g/L peptone, 5 g/L sodium taurocholate, 10 g/L NaCl; pH 8.2±0.2) and incubated at 37°C for 18–20 h. Aliquots were streaked onto TCBS agar and incubated at 37°C for 18–20 h. Yellow colonies were identified as Vibrio spp. and confirmed as *V. alginolyticus* using standard biochemical tests.

Genomic DNA was extracted from an overnight growth of a single isolated colony in LB broth (at 37°C) using the QIAGEN Mini Kit (QIAGEN, Germany), and concentrations were measured with a NanoDrop One (Thermo Scientific, USA). Libraries were prepared with the Illumina Nextera XT Kit (Illumina Inc., USA) and sequenced on the Illumina MiSeq platform using the MiSeq Reagent Kit v2 (2×150 bp paired-end reads) at NSU Genome Research Institute. A total of 692,929 paired-end reads were produced. Reads quality checked with FastQC v0.12.1 (4) and assembled *de novo* using SPAdes v.3.13.1 (5), resulting in a total genome size of 5,242,109 bp (GC content 44.5%). Assembly assessment with Quast v5.3.0 (6) revealed the genome to contain 112 contigs with an N50 value of 271,920 bp, with the largest contig being 540,735 bp.

The genome annotation was carried out using the NCBI Prokaryotic Genome Annotation Pipeline version 6.9 (7), yielding 4,775 genes, including 4,635 protein-coding genes, 3 rRNA genes, and 70 tRNA genes. Assembly quality was assessed using CheckM v1.2.3 (8), indicating 99.6% completeness. Taxonomy was determined using FastANI against RefSeq, showing 98.45% ANI (alignment fraction 89.58%) to the *V. alginolyticus* type strain NBRC 15630 = ATCC 17749 (type assembly GCA_000354175.2), consistent with the NCBI taxonomy check (species_match) for our assembly GCF_044904215.1. Antibiotic resistance genes were identified using ResFinder (2024-03-22; v4.6.0) (9, 10) and the Comprehensive Antibiotic Resistance Database (CARD v4.0.1) via the Resistance Gene Identifier (RGI v6.0.5), retaining hits with ≥90% identity and ≥60% coverage (11). Genes such as *tet(34*) and *tet(35*) (tetracycline resistance), as well as *blaCARB-42* (β-lactam resistance to ampicillin, amoxicillin, and piperacillin) were identified. Virulence factor prediction was predicted using BLASTp (v2.13.0) against the VFDB core data set (Set A, 2023) (E-value ≤1e-5; identity ≥80%) (Table 1) (12). All tools were run with default parameters unless specified.

**TABLE 1:** Identification of Virulence-Associated Genes from VFDB

Gene	Product name	VFDB accession	Coverage	Identity
*vscF*	needle protein	NP_798073	100.0	82.33
*vscI*	inner rod protein	NP_798070	99.71	83.43
*vopR*	Effector protein	NP_798062	99.9	81.06
*vscS*	C-ring protein	NP_798052	100.0	80.9
*vscR*	C-ring protein	NP_798051	99.69	83.2
*tlh*	thermolabile hemolysin	NP_799736	100.0	85.04

This draft genome of *V. alginolyticus* highlights genetic traits relevant to the marine ecosystem. The presence of antibiotic resistance genes and virulence factors underscores the need to monitor marine ecosystems as potential reservoirs for antibiotic-resistant organisms that pose threats to human health.

## Data Availability

The assembled genome sequence has been deposited at GenBank under GCA_044904215.1, with the corresponding Sequence Read Archive (SRA) accession number SRR31100191. The ResFinder, VFDB, and CARD output files have been published on Figshare and are available at (DOI: https://doi.org/10.6084/m9.figshare.28830971.v1).
